# Nivolumab‐induced fatal myocarditis: A case report

**DOI:** 10.1002/ccr3.7306

**Published:** 2023-05-09

**Authors:** Kavya Bharathidasan, Mahmoud Abdelnabi, John Abdelmalek, Jasmine Sekhon, William Butler, Miguel Quirch, Erwin Argueta Sosa

**Affiliations:** ^1^ Internal Medicine Department Texas Tech University Health Science Center Lubbock Texas USA; ^2^ Cardiology Department Texas Tech University Health Science Center Lubbock Texas USA; ^3^ Hematology and Oncology Division, Internal Medicine Department Texas Tech University Health Sciences Center Lubbock Texas USA

**Keywords:** acute heart failure, esophageal cancer, immune checkpoint inhibitors, myocarditis, nivolumab

## Abstract

**Key Clinical Message:**

Baseline assessment and interval monitoring with a careful history, clinical examination, laboratory work‐up, and noninvasive imaging modalities may be beneficial for early detection of immune checkpoint inhibitor‐associated side effects.

**Abstract:**

Previous reports of immune checkpoint inhibitors' cardiotoxic effects include pericarditis, myocarditis, myocardial infarction, ventricular dysfunction, vasculitis, and electrical abnormalities. The authors report a case of acute heart failure caused by nivolumab‐induced cardiotoxicity in a middle‐aged man with advanced esophageal carcinoma with no previous cardiac history or significant cardiovascular risk factors.

## 1 BACKGROUND

Data regarding short‐ and long‐term cardiovascular complications are still lacking yet evidence regarding immune checkpoint inhibitors (ICI)‐induced cardiotoxicity has emerged. Side effects include pericarditis, myocardial infarction, takotsubo cardiomyopathy, ventricular dysfunction, vasculitis, electrical abnormalities, and fulminant myocarditis.^1^ The authors report a case of acute heart failure complicated by cardiogenic shock caused by nivolumab‐induced cardiotoxicity in a middle‐aged man with advanced esophageal carcinoma.

## 2 CASE PRESENTATION

**FIGURE 1:**
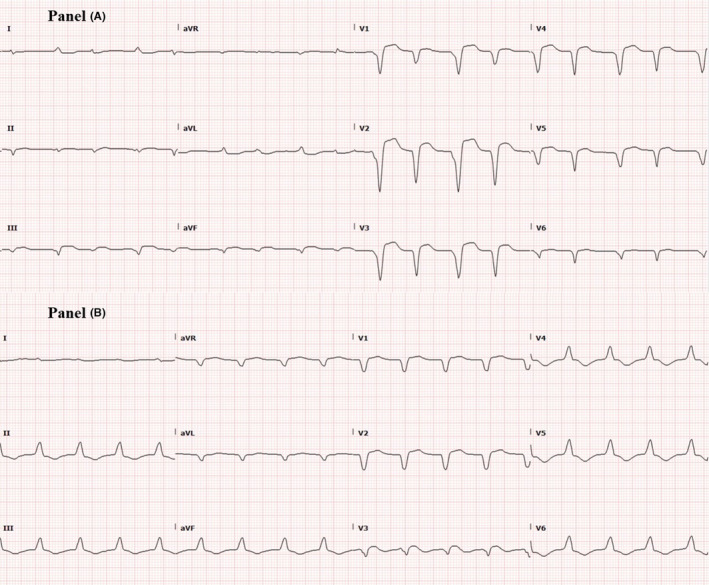
Panel A: ECG showing new‐onset atrial fibrillation with interventricular delay. Panel B: ECG showing new‐onset left bundle branch block.

**FIGURE 2:**
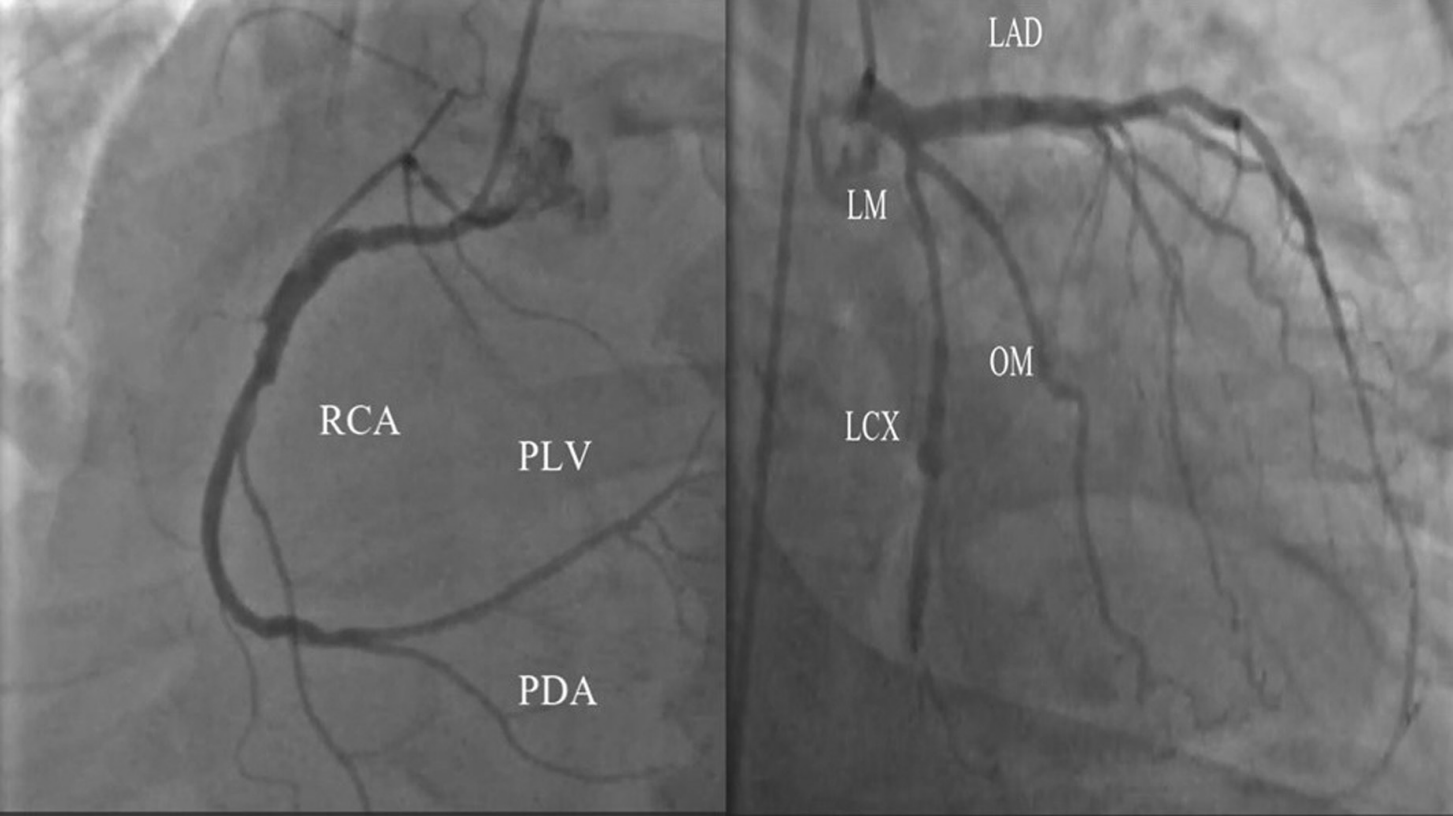
Coronary angiogram showing nonobstructive coronary artery disease.

**FIGURE 3:**
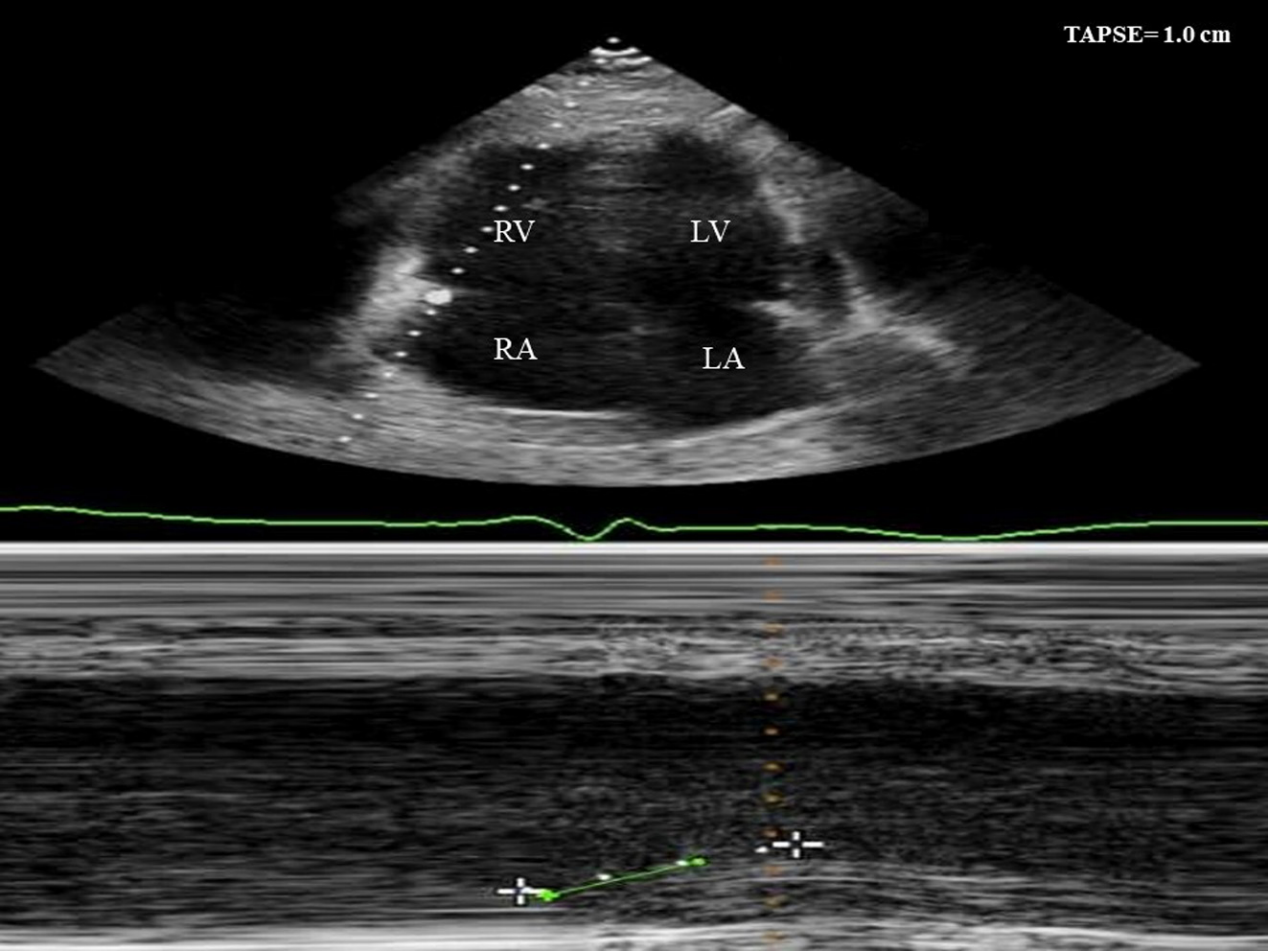
Echocardiography showing biventricular systolic dysfunction with markedly dilated right ventricle (RV) with reduced RV systolic function (tricuspid annular plane systolic excursion (TAPSE) = 1.0 cm).

**FIGURE 4:**
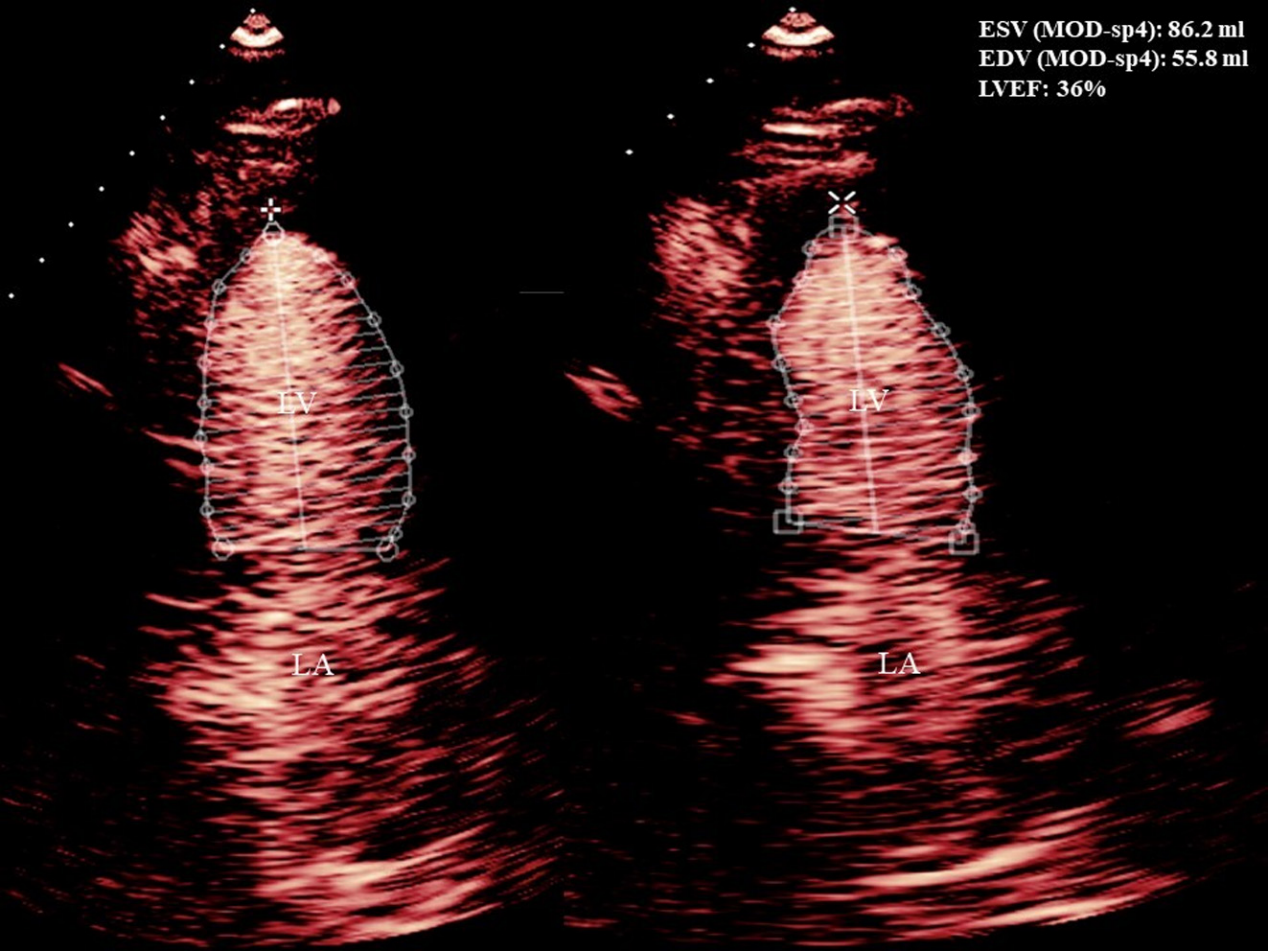
Contrast transthoracic echocardiography showing reduced left ventricular systolic function with an estimated left ventricular ejection fraction of 35%.

A 54‐year‐old male with a history of stage IIIA esophageal adenocarcinoma for which he received neoadjuvant concurrent chemoradiation with six cycles of carboplatin/paclitaxel combination and a total of 50.4 Gray (Gy) in 28 fractions followed by distal esophagectomy and proximal gastrectomy with gastric pull‐through and adjuvant maintenance immunotherapy with nivolumab for 2 months, last dose received 3 days before his presentation. He presented complaining of intractable nausea, vomiting, new onset shortness of breath, and compressing central chest pain with no radiation or referral. He had no cardiovascular risk factors except for previous history of smoking. No recent history of vaccination. On examination, he was distressed, tachypneic (respiratory rate of 20 and oxygen saturation of 92% on room air), and tachycardiac (heart rate of 105 beats/minute, and blood pressure of 110/90 mm Hg). Jugular venous dilatation, bilateral basal crepitations, and tender hepatomegaly were noted. His laboratory workup was significant for elevated renal and liver function, lactic acidosis, and markedly elevated troponin and pro‐B‐type natriuretic peptide (BNP). Serial electrocardiograms (ECG) showed new‐onset atrial fibrillation with interventricular condition delay (Figure 1 Panel A) and follow‐up ECG showed a new‐onset left bundle branch block (LBBB) (Figure 1 Panel B). Based on his new ECG changes and troponin elevation, emergent coronary angiography (CA) was performed showing nonobstructive coronary artery disease (CAD) (Figure 2, Video 1). Urgent echocardiography showed new onset biventricular systolic dysfunction with an estimated left ventricular systolic function (LVEF) of 35% and marked dilated right ventricle (RV) with reduced RV systolic function. (Figures 3 and 4

**VIDEO 1:** Coronary angiogram showing nonobstructive coronary artery disease.

, Video 2). He was promptly started on diuretics, vasopressors, dobutamine and amiodarone infusions, and prophylactic anticoagulation. However, due to his deteriorating condition no further invasive procedures for hemodynamic monitoring, mechanical circulatory support, or endomyocardial biopsy for the assessment of possible myocarditis could be performed aligned with the patient's family's wishes. Viral serology was unremarkable for possible viral etiologies of myocarditis. He was started on pulse steroids (intravenous methylprednisolone 1 gram daily for suspected nivolumab‐induced myocarditis with no significant improvement. Eventually, his family opted to proceed with comfort care measures. He passed away on Day 5 of his hospitalization.

**VIDEO 2:** Echocardiography showing biventricular systolic dysfunction with markedly dilated right ventricle (RV) with reduced RV systolic function (tricuspid annular plane systolic excursion (TAPSE) = 1.0 cm). Contrast transthoracic echocardiography showing reduced left ventricular systolic function with an estimated left ventricular ejection fraction of 35%.

## 3 DISCUSSION

ICI therapy has shown promising results in antitumor activity induction in malignancies such as melanoma, T and B‐cell lymphomas.^2^ Previously reported cardiotoxic effects included pericarditis, myocardial infarction, takotsubo cardiomyopathy, ventricular dysfunction, vasculitis, electrical abnormalities, and fulminant myocarditis.^1^ Several mechanisms of ICI‐related adverse events were proposed such as shared antigen between tumor cells and cardiomyocytes resulting in myocarditis by molecular mimicry.^3^ Animal model studies have shown that cytotoxic T lymphocyte antigen‐4 (CTLA‐4) deficiency can cause myocarditis due to uncontrolled lymphocytic infiltration.^1^ Since programmed cell death protein 1 (PD‐1) is known to have cardio‐protective effects by tissue inflammation inhibition and myocardial damage prevention, animal studies have shown that PD1−/− cytotoxic T lymphocytes (CD8+) T cells compared to PD1+/+ CD8+ T cells can result in fulminant myocarditis.^4^ Also, PD‐1 deficiency was associated with autoimmune dilated cardiomyopathy.^5^ Therefore, it is proposed that ICI likely lowers the T cells activation threshold against myocardial self‐antigens.^6^ Diagnostic strategies should aim not only to confirm the diagnosis but also to rule out other common cardiac causes of similar clinical manifestations (illustrated in Table 1). High initial and at‐discharge troponin were shown to confirm the diagnosis and evaluate the overall prognosis of myocarditis. Unlike troponin, elevated BNP levels were not associated with a worse prognosis.^3^ ECG changes such as new PR interval prolongation, atrioventricular block, ventricular arrhythmias, frequent premature ventricular complexes, ST depression, or diffuse T‐wave inversions can suggest myocarditis. Data did not support troponin or ECG use for surveillance given that the incidence of myocarditis is rare.^3^ Baseline echocardiography before initiation of any potential cardiotoxic therapy is recommended. Echocardiographic signs associated with ICI myocarditis include LVEF changes, diastolic function, new wall motion abnormalities, or pericardial effusion. Echocardiography can also be used to monitor treatment response.^3^ Cardiac magnetic resonance imaging (CMR) is emerging as a validated noninvasive modality to diagnose myocarditis. Diagnostic criteria include main criteria (two of two) including signs of myocardial edema as abnormal findings in T2 mapping or T2‐weighted images or signs of nonischemic myocardial injury as abnormal findings on T1 mapping, late gadolinium enhancement (LGE), or extracellular volume fraction while supportive criteria include signs of pericarditis as pericardial effusion or abnormal LGE/T2 or T1 findings in the pericardium or left ventricular systolic dysfunction as regional or global wall motion abnormalities.^7^ Endomyocardial biopsy is the gold standard for the diagnosis of myocarditis. Histologic criteria for diagnosis require inflammatory infiltrate (global or focal with patchy disease) and myocardial necrosis.^8^ Although CA does not have a role in diagnosing myocarditis, it is often performed to rule out CAD as a cause of similar clinical presentations, elevated biomarkers, ECG changes, or imaging abnormalities.^3^ ICI‐induced myocarditis treatment is based on ICI discontinuation and prompt initiation of pulse steroids followed by a steroid taper. Other options for treatment include immunoglobulin, anti‐thymocyte globulin, plasmapheresis, and mycophenolate as previously reported in case reports and small case series. No current data exists regarding the safety of ICI restart after an episode of successfully treated ICI‐related myocarditis.^3^ Recently emerging data demonstrated an increased incidence of atherosclerotic cardiovascular (CV) complications in ICI‐treated patients due to accelerated atherosclerosis.^9^ A matched cohort study showed that there was a threefold higher risk of composite and individual components of atherosclerotic CV events (myocardial infarction, coronary revascularization, and ischemic stroke) and a > 3‐fold higher rate of progression of total aortic plaque volume with ICI use which might be explained by low‐grade arterial wall inflammation and plaque progression which was independent of classical CV risk factors, or cancer type. Concomitant use of statins or corticosteroids has been shown to attenuate atherosclerotic plaque progression.^10^ The authors report a case of nivolumab‐induced acute heart failure and cardiogenic shock in a middle‐aged man with advanced esophageal carcinoma with no previous cardiac history or significant cardiovascular risk factors. His clinical presentation was consistent with fulminant myocarditis which should be considered in patients without typical cardiovascular risk factors presenting as more common conditions such as acute coronary syndromes (ACS) or de novo acute heart failure, patients with electric instability, or rapidly evolving conduction abnormalities such as widening of the QRS complex or PR prolongation. He initially presented with new onset LBBB and elevated cardiac biomarkers, yet CAD was excluded by CA. Clinical, and laboratory and imaging modality showed signs of acute biventricular systolic dysfunction with basal crepitations, dilated neck veins, tender hepatomegaly, elevated BNP, renal and liver function tests. Several patterns of electrical instability were noted in his ECGs. The temporal relation between nivolumab and the abrupt onset of his symptoms led to the diagnosis of nivolumab‐induced fulminant myocarditis. Ideally, CMR or endomyocardial biopsy would have been confirmatory, but given his rapidly deteriorating condition and his family's request, neither was done.

**TABLE 1:** Diagnostic criteria for clinically suspected myocarditis.

Clinical presentations
Acute chest pain, percarditic, or pseudo‐ischemic New onset (days up to 3 months) or worsening of: Dyspnea at rest or exercise, and/or fatigue, with or without left and/or right heart failure signs Subacute/chronic (>3 months) or worsening of dyspnea at rest or exercise, and/or fatigue, with or without left and/or right heart failure signs Palpitations, and/or unexplained arrhythmia symptoms and/or syncope, and/or aborted sudden cardiac death Unexplained cardiogenic shock
Diagnostic criteria
ECG/Holter/stress test features
Newly abnormal 12 lead ECG and/or Holter and/or stress testing, any of the following: I to III‐degree atrioventricular block, intraventricular conduction delay (widened QRS complex), bundle branch block, sinus arrest, or asystole ST/T wave changes (ST elevation or non‐ST elevation, T wave inversion) Low voltage, abnormal Q waves Frequent premature beats, supraventricular tachycardia, atrial fibrillation, or ventricular tachycardia or fibrillation
Myocardiocytolysis markers
Elevated cardiac troponin I (TnI) and troponin T (TnT)
Functional and structural abnormalities on cardiac imaging (echocardiography/CA/CMR)
New, otherwise unexplained LV and/or RV structure and function abnormality (including incidental findings in apparently asymptomatic subjects): Regional wall motion or global systolic or diastolic function abnormality, with or without ventricular dilatation, with or without increased wall thickness, with or without pericardial effusion, with or without endocavitary thrombi.
CMR tissue characterization
Edema and/or late gadolinium enhancement (LGE), LGE of classical myocarditis pattern

*Note*: Clinically suspected myocarditis if ≥1 clinical presentation and ≥1 diagnostic criterion from different categories, in the absence of (1) angiographically detectable coronary artery disease (coronary stenosis ≥50%); (2) known pre‐existing cardiovascular disease or extra‐cardiac causes that could explain the syndrome (e.g., valve disease, congenital heart disease, hyperthyroidism, etc.). Suspicion is higher with a higher number of fulfilled criteria. If the patient is asymptomatic ≥2 diagnostic criteria should be met.

## 4 CONCLUSION

Although the incidence of cardiac complications with ICI seems to be rare, baseline assessment and interval monitoring with a careful history, clinical examination, laboratory work‐up, and noninvasive imaging modalities may be beneficial for early detection of serious side effects such as fulminant myocarditis, thus allowing prompt discontinuation and immediate initiation of high‐dose steroids for improved outcomes.

## AUTHOR CONTRIBUTIONS

**Kavya Bharathidasan:** Writing – original draft; writing – review and editing. **Mahmoud Abdelnabi:** Validation; writing – original draft; writing – review and editing. **John Abdelmalek:** Resources; validation; visualization. **Jasmine Sekhon:** Resources; validation; visualization. **William Butler:** Resources; validation; visualization. **Miguel Quirch:** Supervision; validation; visualization; writing – review and editing. **Erwin Argueta Sosa:** Supervision; validation; visualization; writing – review and editing.

## CONFLICT OF INTEREST STATEMENT

The authors have no conflict of interest to declare.

## FUNDING INFORMATION

None.

## CONSENT

The authors have obtained written informed consent from the patient's medical power of attorney to publish his medical history/course and case details in accordance with the journal's patient consent policy.

## Data Availability

The data areis available for sharing.
